# Locating crisis

**DOI:** 10.1177/27541258251384303

**Published:** 2025-10-09

**Authors:** Cristina Temenos

**Affiliations:** 1 5292 University of Manchester, UK

**Keywords:** Crisis, crisis policy-making, conjuncture, urban politics, urban health

## Abstract

In this short intervention, I reflect on how I have used the concept of crisis in my work over the past 15 years. I argue that a spatial and pluralised understanding of crisis is essential to make sense of the political, material, and discursive stakes of contemporary urbanisation, and offer a reading of crisis that sits between the eventful and everyday. I outline ways in which urban crisis can be understood apart from, yet constitutive of a concept of crisis ‘writ-large’, and demonstrate through brief empirical examples how locating crisis conjuncturally defines its relationship to the state and social reproduction of and in the city.

## Introduction

Cities today are facing a series of intersecting crises for which they are increasingly ill-equipped to respond, given that urban societies are unable to rework their political configurations to overcome or reconcile with neoliberal logics, breaking the social contract, and disavowing any understanding of the public good. The framing of crisis itself, both as a situational diagnostic and empirical descriptor, is one of the reasons for this impasse. If we want cities to be able to face the increasingly rapid pace of emergencies amidst geographic unevenness and ongoing, historically embedded inequality, then it becomes increasingly important to pluralise our understanding of crisis. By examining what sort of intellectual project is necessary to theorise the relationship between crisis, cities, and urbanisation, a pluralised understanding of crisis is spatialised, contingent, and predicated on understanding crisis as a political concept as much as it is an analytic. Pluralising an understanding of crisis necessitates a focus on the materialisation scales, actors, objects, and urban forms through a relational–conjunctural lens. This, in turn, highlights relationships between their existence in multiple autonomous worlds and how these worlds are simultaneously emergent and interconnected. This essay focuses on understanding the relationship between crisis and the urban, as opposed to other types of spatialised crises (e.g. national, regional, and global) or crises writ large, to demonstrate the intertwining of scales that come together to create conditions for crisis. In this sense, a crisis might be understood as an assemblage (Baker and McGuirk, 2017; McCann and Ward, 2013; McFarlane, 2011) or conjuncture (Davidson and Ward, 2024; Doucette, 2025; Hart, 2024; Lorne et al., 2024). A focus on urban crisis allows for an analysis of contingencies across spaces and scales to reveal the coming together of lived political and everyday realities of ongoing hardship and change.

Invoking crisis in contemporary discourse and practice invokes urgency, exceptionalism, and imperatives to act. It offers a compelling prognostic for the current zeitgeist. We hear it on the news, on social media, in scholarship, and through everyday discourse. These days, crisis is seemingly everywhere. However, if crisis is everywhere and in everything, it risks a depoliticisation, explaining everything and nothing all at once. While crisis scales from the intimate to the global, to borrow Pratt and Rosner's (2012) phrasing, *urban crisis* suggests that a focus on the embodied spatial elements of crisis is essential to give it meaning and materiality as a concept. Crisis, broadly speaking, is often treated as episodic, a one-off culmination of processes or an event. Focusing on urban crisis demonstrates how crisis becomes normalised and embedded, both spatially and temporally, as part of the everyday experience.

Locating crisis in the urban demonstrates how it is relationally reconfigured through discourses, practices, politics and material reworking of urban space, as well as how, in turn, it acts as a mobilising force. Not only is it essential to think through how we can understand crisis as it unfolds *through* space, but it is equally important to understand crisis *as* a space. Therefore, spatialising understandings of crisis fights against the seeming banality of crisis invocations in contemporary scholarship, urban and otherwise (Fredriksen, 2025; Temenos, 2025). It makes clear the work that crisis does in mediating relational connections, and thus its relation to cities, urbanisation, and urban life. Moving away from a dualistic understanding of crisis as either a rupturing event or ongoing state towards a pluralistic understanding of crisis spatialities uncovers other ways of thinking about crisis, demonstrating it as a historicised, political, and mobilising force, enabling certain discourses and material futures to emerge over others. In this sense, recent interventions on conjunctural approaches have proved a useful way of bringing together relational approaches with methodological comparison (Hart, 2018; Ward, 2010).

Lorne et al. (2024: 502) note that conjunctural analysis at its most potent is a way of raising politically strategic questions about the various struggles, antagonisms, and forces that converge in moments of crisis, with the aim of identifying possibilities for alternative political directions. As a method, conjunctural analysis provides a way to make sense of the present in all its complexity and multiplicity. This approach is concerned with how different crises, tensions, and contradictions become condensed in a specific historical moment (Clarke, 2010; Hall and Massey, 2010; Hart, 2024). Hall et al. (1978[2017]) remind us that futures are never guaranteed; there is no way to know in advance which struggles or forces will come to shape a given conjuncture. More than simply recognising that multiple dynamics are in play at once (Clarke, 2018), conjunctural thinking pushes us to determine which connections, both analytical and political, are most significant, and where there might be openings for social and political transformation (Lorne et al., 2024). Hart (2024) and, more recently, Doucette (2025) have argued that it can be found in a global conjunctural framing, which is useful in negotiating the multiple temporalities that stitch together processes operating across scales in particular or situated configurations.

What can it mean for the urban political, particularly in times of increasing and intersecting crises? The value of a conjunctural framing is that it provides a method for spatialising urban relations of power, taking seriously the processes that go into making up urban space and the relationship between the state and everyday life. Heeding Clarke's (2018) caution not to reduce the conjuncture to a matter of periodisation, but rather to approach it as dealing with the different temporalities that come together and are condensed in the conjunctural moment, he argues that

It is precisely in the conjunctural *entangling* of different dynamics that we can find the conditions in which these different dynamics and their distinctive temporalities come together in complex articulations as they groom, condition, interrupt and unsettle one another … however, is the importance of thinking of this presence of different temporalities as taking the form of active and intense condensation rather than a passive or indifferent coexistence (Clarke, 2018: 205).

Understanding the mobility and speed of those temporalities as they intersect is an important aspect of a conjunctural framing. Beyond the temporal, conjunctural analysis offers us a method to situate urban crises, whether housing, policing, infrastructure, or climate, not as isolated failures, but as symptoms of broader shifts and contradictions. It pushes us to ask: who gets to name the crisis, and what and where are the consequences of this naming? Crisis is spatialised through histories of city-making, asking who is saved and who is left adrift during times of crisis? In addition, in what ways do crises reshape processes of urbanisation and the built environment? Finally, the crisis is spatialised through practices of resistance. What actions, arguments, and political solidarities arise as crisis discourses and realities unfold? How do they transform and create new urban arrangements?

Cities constitute pivotal sites where crisis is both produced and lived, emerging from particular assemblages of density, proximity, and social encounter. If a crisis is spatially differentiated, then the concept of an urban crisis demands analytic attention in its own right. While cities are increasingly marked by the intersection of multiple, overlapping emergencies, from housing precarity and infrastructural breakdowns to climate-induced shocks, pandemics, and escalating urban violence, the effects of urban crisis are not confined to the borders of cities as discreetly bounded entities. These phenomena converge to frame our present conjuncture as an age of crisis, in which ecological, economic, political, and social crises proliferate with increasing rapidity and interconnection. Within this conceptualisation of urban crisis, the urban acts as both a political and generative force, one through which crisis is mediated (cf. Barnett, 2014; Beveridge and Koch, 2022, 2024; Boudreau, 2016; Peake et al., 2021; Simone and Pieterse, 2017).

Such intensified crisis tendencies can be situated within the broader structural dynamics of contemporary capitalism and the uneven development of state capacities. Extended periods of global austerity have not only eroded architectures of social reproduction but have also compromised the ability of communities and local governments to absorb and respond to accumulating shocks. Furthermore, crises are not only outcomes of economic conditions, they are shaped by the circulation and implementation of particularly urban policy models, which often reinforce structural inequalities and political ideologies. As I show below, crisis response mechanisms, particularly in the area of public health and drug policies, are politically contingent and unevenly distributed. This can paradoxically reflect logics of neoliberal urban governance while working toward achieving equitable and just access to health for all (McCann and Temenos, 2015; Temenos, 2017; Temenos and McCann, 2013). The urban built environment, in its material and social form, is both a terrain and a tool of capital accumulation, where crises function simultaneously as moments of rupture and reconfiguration (Davidson and Ward, 2018; Harvey, 1989; Jessop, 2012).

In the next section, I briefly set out how urban scholars have engaged with concepts of crisis to argue for a spatial approach to urban crisis at the interstices of the eventful and ongoing (cf. Temenos, 2022, 2025). I then draw on brief examples from my own research to demonstrate the understanding of urban crisis as a multi-dimensional, relational and mobilising force, shaping and shaped by cities, urban life, and urban politics. I conclude by arguing that a spatial understanding of urban crisis transforms the concept of crisis into an essential analytic to understand the political, material, and discursive stakes of contemporary urbanisation.

## Pluralising urban crisis

Crisis can be conceptualised variously as an event that reorders spatial and political relations (Agamben, 2008; Roitman, 2020); as an ongoing condition that saturates everyday life and political arrangements (Berlant, 2011; Harris et al., 2019); and as a contingent conjuncture of the ongoing, the eventful, and the everyday (Dimitrakou and Ren, 2025; Temenos, 2022, 2025). Understanding urban crisis thus requires an engagement with these multifaceted temporalities and the political–economic structures that produce them, highlighting how crisis is both a product and a process of urbanisation and contemporary life in cities.

Urban crisis is tangible, material, and territorial. It can refer to a range of specific conditions of urban form, physical, political and social infrastructures. For example, ageing and poor-quality housing stock, unstable municipal budgets and urban governance under economic austerity, declining industrial bases, rising welfare rates in response to growing social and economic inequalities, rolled back social and public health services, dilapidated parks and reduced green space and biodiversity, poor air quality. Urban crisis tends to manifest as an outcome of particular conjunctures of national and regional policies, global capital and migratory flows, and localised inhabited spaces. This has an embodied effect, as the culmination of urban crisis contributes to poorer quality of life, well-being, and health outcomes, including contemporary urban afflictions such as rising rates of respiratory illness, mental health emergencies, increased substance use, increased rates of death by suicide, and early deaths by preventable conditions. Further, urban crises then, crises of and in the city, often lead to political crises such as rising right-wing backlash, political unrest and/or disengagement in democratic processes in the city, as well as beyond (cf. Davies, 2024; Doucette, 2025; Fainstein and Novy, 2025).

While urban crises may indicate rupture, so too can they reproduce urban regimes of power. Focusing on crisis only as a point of departure risks a focus on spectacular urban failures while obscuring the more common slow-structural violence of the state. Yet working with the concept of urban crisis can also provide a way into thinking through the material and political consequences of failure in urban spaces (Lauermann and Temenos, 2022). A crisis can be understood as an entangled moment where economic, political, cultural, and social processes converge. Failure stemming from and in the face of crisis can be both intentional and unexpected. Jones and Ward (2002) outline how approaches in urban economic geography often neglect the political stakes of crisis, arguing that a closer analysis of the state is necessary to understand the ways in which crisis works to reproduce itself and to reproduce economic logics of governance, what they frame as the ‘crisis of crisis management’ for urban policy. Moreover, the ways in which the state is conceptualised in urban studies shape analytical and empirical attention to how crisis is framed in crisis scholarship. Attention to the relationships between the state and social reproduction vis-à-vis crisis can draw out a pluralised understanding of the political framings and material consequences of crisis (Temenos, 2017, 2022). It further illuminates the complex scalar politics that make up urban geographies, challenging urban scholars to theorise the relationship between the state, the city and urbanisation in ways that challenge neatly defined narratives of urban development and contemporary urban life (Simone and Castán Broto, 2022; Temenos and Ward, 2025).

Urban economic geography has traditionally focused on fiscal downturn and deindustrialised decline (Harvey, 1989; Jessop, 2012), yet the analysis must stretch further. Drawing from Hall and Massey's (2010) framing of urban crisis as a ‘complex moment’, it is possible to disassemble how cities are both shaped and undone by interlocking systems of power. For example, the materiality of crisis, as rendered in visualisations such as the foreclosure maps of the 2007 Financial Crisis, elucidates the lived entanglement of housing markets and global capital (Fields and Hodkinson, 2018; Shelton, 2022). Such tools also remind us that crisis failures are never only about capital and the market; they reside in bodies, infrastructures, and everyday life, demanding that we look beyond abstract systems toward the socially reproductive forces that hold city life together.

Urban scholarship on crisis has foregrounded relational and conjunctural approaches to crisis. In *Policing the Crisis*, Hall et al. (1979) traced the racialised grammar of state violence, revealing crisis not as a singular event but a Gramscian conjuncture, a gathering of elements in places where systems endure even as they mutate. Harvey's (1982) formative analysis of capital in the urban landscape invokes crisis as a mobilising force, initiating the creative destruction of ongoing urban development at the expense of place. Berlant's (2011) notion of the ‘crisis-ordinary’, while not specifically attending to the urban condition, obliges a gaze on the slow, attritional violences that structure daily life. They focus on the mundane erosions of care, time, and capacity. Whether seen as an ongoing condition or as a rupture, crisis has a spatio-temporal force, punctuated by moments that may open political possibilities. At these interstices, between event and process, urban crisis becomes a site of struggle, of potential, and of imaginaries of emergent urban futures. It is located at these conjunctures that new political entanglements may be forged.

## The politics of crisis governance

Drawing on my own research, I explore these themes through the relationship between the state and everyday life, seeking out an everyday proper politics that takes seriously the multifaceted spatialities of cities and the ways people, communities, and governments work to create more equitable and just spaces and conditions for living (Temenos, 2016, 2017, 2022, 2024). For example, ‘crisis policy-making’ is one conceptual tool to interrogate how governance unfolds amid layered crises (Temenos, 2022). This mode of policy-making and, crucially, of politics is shaped by four interwoven dynamics: speed, opacity, revanchism, and experimentation. Crisis accelerates decision-making; what once seemed impossible becomes law overnight. Yet the urgency of action often shrouds decisions in obscurity, justified by the rhetoric of emergency. Such opacity frequently conceals revanchist motives, and too often, the outcomes are policies and programmes that deepen inequality and marginalise those dissenting or vulnerable. However, crisis also opens space for experimentation, a double-edged condition. While many experiments reinforce neoliberal governance, some offer glimpses of alternative solidarities and urban care. In these moments, cities become spaces in which the potential of alternative urban futures becomes clear, places where cracks in the current order allow for something else to take root.

Geographies of urban health have long been one of the most embodied fields of interrogating the politics of the state–everyday life nexus. Health crises are acutely felt at a personal level and within communities. They move quickly, and when scaled up, often have long-lasting effects on people and places. The AIDS crisis of the 1980s, which still reverberates today, shifted urban healthcare, urban politics, and transformed urban landscapes through long-standing struggles around care provision. Wrapped up in socio-legal debates about behaviours such as drug use and homosexuality that were (and in many cases still are) criminalised, the AIDS crisis precipitated healthcare practices emerging from mutual aid: harm reduction for safer sex initiatives, needle exchange, wound care, and eventually access to potentially life-saving medications. These practices were first and foremost practical, yet they were also inherently political, based on an understanding that overrode legal frameworks criminalising certain behaviours and motivated through radical democratic understandings of equity and humanity in accessing care.

While mutual aid surrounding the provision of sterile syringes, for example, was a practical act of resistance and mutual aid in the early 1980s and 1990s, it quickly became politicised by the arrest of healthcare workers, activists and citizens such as the Needle Eight, members of the HIV/AIDS activist group ACT UP! arrested for setting up a public needle exchange in the Lower East Side of New York City in 1990. The 1991 court ruling exonerated the eight charged and helped set a legal precedent for acts of resistance to provide medically necessary care during times of crisis, while also contributing to a legal geography that set out a landscape for the legalisation of syringe exchange. Thus, it moved such practices borne of crisis from back alleys and church basements into front-line health services, visible healthcare outreach, and community pharmacies.

More recent ways that access to healthcare in cities has been shaped through financial crisis and mutual aid can be seen in the social clinics and pharmacies that emerged in urban centres, from Athens, Greece, to Santiago, Chile. The social clinics that emerged operate on two different models. The Greek model of social clinics and pharmacies was and remains based on mutual aid and volunteers. In the wake of the 2007 Global financial crisis and the 2011 austerity directives, Greece introduced a policy restricting access to national health insurance for the unemployed to 12 months, effectively severing universal health coverage for nearly a quarter of the population. These were individuals disproportionately impacted by the deep structural unemployment of a collapsing economy, and for whom private healthcare was simply inaccessible. Simultaneously, from 2009 to 2012, public health expenditures in Greece declined by 24% leading to closures and restricted hours in many state facilities (Goranitis et al., 2014). This precipitated the rapid outmigration of health professionals to other European Union countries, stifling domestic care capacity. The result was a contraction of access to care, exacerbated by a reduction in the staff required to deliver it.

What emerged, however, in this enforced absence of institutional support was not just a stopgap (though it was), but also a radical reconfiguration of care practices. Across cities in Greece, and most notably in Athens, solidarity health clinics and pharmacies emerged. These clinics, operated entirely by volunteers and positioned explicitly outside the domain of state institutions, political parties, or international non-governmental organisations, responded to a widening care vacuum. Located in both urban squats as well as municipal buildings, which cities voluntarily let remain in use, they provided a range of services from preventive and chronic care, vaccinations, and the distribution of prescription medicines. The clinics formed a distributed network of mutual aid.

At their height, estimates suggested between 92 and 137 solidarity clinics operated in cities nationally, with nearly 50 in Athens alone. Their governance structures reflect a deliberate politics of horizontality with decisions typically made through general assemblies that include both volunteers and patients (Evlampidou and Kogevinas, 2019; Temenos, 2022). The emergence of solidarity clinics can be read not merely as a humanitarian response to a crisis of care but also as an urban political intervention, a grassroots infrastructure of care permitted (and perhaps tacitly encouraged) by a state seeking to mitigate the consequences of its own withdrawal. These clinics are spatial articulations of political resistance and collective care in the face of ongoing crises precipitated by the structural violence of austerity.

While many of the clinics have since closed due in part to the Greek government's clearance of the urban squats in 2019, and in part to the Greek state's restitution of health insurance, the social pharmacies attached to them have remained a key feature of urban health systems in Greek cities. Ongoing austerity means that many, even those with state health insurance, cannot afford the 20% co-pay on medications, and the social pharmacies remain a key site of access for not only essential medicine, but also healthcare and medical advice. Here, the history of crisis has been written into the urban fabric of Athens, the geography of health and care still shifting amidst the ongoing economic and social crises that affect Greek urbanites.

In Chile, the farmacia popular model is another direct response to converging crises. Emerging in the Santiago metropolitan region in the aftermath of a scandal that exposed widespread pharmaceutical price-fixing, the initiative was not simply a novel public service but a localised intervention into a broken system. In 2015, amidst mounting public anger, rising economic inequalities, and deepening social fragmentation, the first municipal pharmacy opened in Recoleta, a working-class city just north of Santiago's centre, which boasted a government that had adopted a new municipalist framework of operation. This was a moment defined by distrust in market institutions, a collapsing legitimacy in central governance, and spiralling inequality. These conditions saw the discrediting and eroding of the state's capacity to ensure health access.

Farmacia populares provides medicines at 40% to 80% below market prices. Chile, at the time, had some of the highest medication costs in Latin America. In 2019, the average Chilean household was spending 38.5% of its income on medicine (Suis, 2022). In this sense, the farmacia popular was not just a solution to scarcity, but a policy response to a crisis in affordability, made possible by municipal actors stepping into the void left by neoliberal deregulation and national inaction. While initially driven by an initiative of Recoleta's Communist mayor, Daniel Jadue, the model's uptake quickly transcended partisan lines. Today, over 212 farmacias populares operate across 170 municipalities, including longstanding electorally conservative municipalities. This diffusion suggests that crisis responses at the urban scale often invite pragmatic solidarities, even amongst ideologically fragmented territories.

The farmacia popular is more than a band-aid. It represents an urban health infrastructure emergent from crisis and shaped by experimentation. It draws on municipal legal authority, rather than extra-legal mutual aid, to operate. Two such different models of social pharmacies, which emerged more or less concurrently, across Athens and Santiago, complicate the binary we often see in narratives of urban crisis between state-led and grassroots responses: the Chilean model is a state-enabled, municipally embedded form of collectivised care, demonstrating how crisis can produce new forms of statecraft at the urban scale.

## Conclusion

Crisis is not a fixed entity; rather, it is spatially constituted. That is, it is lived and felt differently across interconnected geographies. It is *located* not at a single site but within spatial relations that shape power, economy, and everyday life. Crisis mediates relational connections by distorting their conditions of encounter: it simultaneously compresses as well as extends time, amplifying inequality. Yet, it can also encourage a topological reconfiguration of solidarities and spaces across distance and within urban environments. Crisis is both the expression of how, and a mode through which space becomes contested terrain where futures, urban and otherwise, may be perpetually deferred yet always produced in relation to the current contemporary moment. A conjunctural approach to cities, and indeed of health in cities, brings a fuller relational understanding of how urban care shifts and mutates over time in relation to crisis. Conjunctural thinking on urban crisis shows how discourses and practices are mobilised in specific ways that change legal frameworks, the make-up of urban built environments, and urban culture and urban life.

I have argued in this essay that urban crisis, likewise, is not a singular event or breakdown, but rather it tends to emerge as a diagnostic that elucidates historically embedded processes leading to inequalities. Its invocation is often to name moments when urban systems appear to fail, and thus is inherently political. The acute crisis moment, whether it be housing shortages, rising inequalities, or disease outbreaks, is symptomatic of the wider ongoing conditions, the slower structural violences of the state and social life (cf. Lees and Hubbard, 2022). Urban crisis is geographically uneven, not only between cities but within them. Tied to the crisis of one neighbourhood is the wealth of another. Landscapes of urban care shift and mutate over time in relation to crisis. Moreover, crisis mobilises discourses and practices that change legal frameworks, the make-up of urban built environments, and urban culture and urban life. Indeed, as Katsikana (2024) notes, crisis is deeply imbricated in the city, transforming it over and across shifting registers and timescales. The temporal evolution of crises’ material and discursive forms, effects, and framings gives crisis a morphological element as well as its political use as a tool to narrate, mobilise, and enact certain urban futures over others.

The relationship of crisis to urban theory and urban geography is thus a way of describing entangled geographies of injustice, where historical legacies and contemporary materialities shape spatial politics, social reproduction and survival. It is not only about points of failure (Temenos and Lauermann, 2020), but crisis also brings into focus those whose lives are made precarious, where, and to what end. This analysis, then, is not just about urban crisis’s location in the city, but how the city functions as a site and symbol of broader structural tensions. If the urban is a dense convergence of infrastructures, economies, bodies, histories, and imaginaries where contradictions of capitalism, colonial legacies, and social reproduction are concentrated and made visible; then urban crisis emerges when these contradictions become (or are perceived to be) untenable: housing becomes unaffordable, transit systems fail, policing becomes hyper-militarised, climate shocks expose infrastructural inequality. However, the urban is also relational, connecting distant places through financial flows, supply chains, migration, policy experiments, together with its embedded and localised social, political and economic histories.

So, how then to operationalise urban crisis as an analytic? Following Simone and Castán Broto (2022), I suggest that we cannot simply stop where Massey (2011) left off, at approaching the city, and in this case, crisis, as constitutive of relational flows. It is essential to build on her thinking to instead take seriously ‘the multiple ways in which spaces of relationality are also constituted in relation’ to crisis (Simone and Castán Broto, 2022: 773). Urban crisis as an analytic draws out tensions which may be irreconcilable. Capitalist crisis perpetuates the redevelopment of urban cores, producing financialised investments rather than homes, while simultaneously displacing existing communities. New urban forms of living reduce sociality in the city, thus cutting off spaces and opportunities for community building or radical democratic initiatives, organising or resistance. Studying urban crisis demonstrates that this familiar urban legend is just that, a tall tale, but not one which should foreclose an understanding of more communal or progressive urban life and urban change. Taking urban crisis as an analytic demonstrates how the urban becomes a lived geography through which not only crisis, but also urban futures are made, experienced, and contested.
